# WARS1, TYMP and GBP1 display a distinctive microcirculation pattern by immunohistochemistry during antibody-mediated rejection in kidney transplantation

**DOI:** 10.1038/s41598-022-23078-z

**Published:** 2022-11-09

**Authors:** Bertrand Chauveau, Antoine Garric, Sylvaine Di Tommaso, Anne-Aurélie Raymond, Jonathan Visentin, Agathe Vermorel, Nathalie Dugot-Senant, Julie Déchanet-Merville, Jean-Paul Duong Van Huyen, Marion Rabant, Lionel Couzi, Frédéric Saltel, Pierre Merville

**Affiliations:** 1 Department of Pathology, Pellegrin Hospital, Bordeaux University Hospital, Place Amélie Raba Léon, 33000 Bordeaux, France; 2 University of Bordeaux, CNRS, ImmunoConcEpT, UMR 5164, 146 Rue Léo Saignat, 33000 Bordeaux, France; 3 Department of Nephrology, Transplantation Dialysis, Apheresis, Pellegrin Hospital, Bordeaux University Hospital, Place Amélie Raba Léon, 33000 Bordeaux, France; 4 University of Bordeaux, Oncoprot Platform, TBM-Core US 005, 33000 Bordeaux, France; 5 University of Bordeaux, INSERM UMR1312, BoRdeaux Institute of onCology (BRIC), 33000 Bordeaux, France; 6 Laboratory of Immunology and Immunogenetics, Pellegrin Hospital, Bordeaux University Hospital, Place Amélie Raba Léon, 33000 Bordeaux, France; 7 University of Bordeaux, Platform of Histopathology, TBMCore - INSERM US005 - CNRS UAR 3427, 33000 Bordeaux, France; 8 INSERM U970, Paris, France; 9 Department of Pathology, Necker Hospital, Assistance Publique – Hôpitaux de Paris (AP-HP), Paris, France; 10 INSERM U1151, Paris, France

**Keywords:** Diagnostic markers, Kidney diseases, Immunopathogenesis, Translational immunology, Allotransplantation, Machine learning

## Abstract

Antibody-mediated rejection (ABMR) is the leading cause of allograft failure in kidney transplantation. Defined by the Banff classification, its gold standard diagnosis remains a challenge, with limited inter-observer reproducibility of the histological scores and efficient immunomarker availability. We performed an immunohistochemical analysis of 3 interferon-related proteins, WARS1, TYMP and GBP1 in a cohort of kidney allograft biopsies including 17 ABMR cases and 37 other common graft injuries. Slides were interpreted, for an ABMR diagnosis, by four blinded nephropathologists and by a deep learning framework using convolutional neural networks. Pathologists identified a distinctive microcirculation staining pattern in ABMR with all three antibodies, displaying promising diagnostic performances and a substantial reproducibility. The deep learning analysis supported the microcirculation staining pattern and achieved similar diagnostic performance from internal validation, with a mean area under the receiver operating characteristic curve of 0.89 (± 0.02) for WARS1, 0.80 (± 0.04) for TYMP and 0.89 (± 0.04) for GBP1. The glomerulitis and peritubular capillaritis scores, the hallmarks of histological ABMR, were the most highly correlated Banff scores with the deep learning output, whatever the C4d status. These novel immunomarkers combined with a CNN framework could help mitigate current challenges in ABMR diagnosis and should be assessed in larger cohorts.

## Introduction

Short-term renal allograft survival has considerably increased over past decades, thanks to improvements in immunosuppressive strategies. In contrast, long-term allograft survival has not increased proportionately and is now a major issue^1^. The leading cause of kidney allograft failure is antibody-mediated rejection (ABMR), considered to be involved in about two-thirds of cases^2^. Antibody-mediated rejection is primarily an endothelial disease mediated by donor-specific antibodies (DSA), which target human leukocyte antigens (HLA) or non-HLA antigens. DSA binding to endothelial cells leads to recruitment of inflammatory cells and injuries (from activation to cell lysis), resulting in histological lesions of microvascular inflammation: glomerulitis and peritubular capillaritis. These two lesions are graded from 0 to 3 (g and ptc scores, respectively), according to the 2019 Banff classification^3^. Moreover, DSA can activate the classical complement pathway and lead to C4d deposits on peritubular capillaries, which can be revealed by immunohistochemistry in a kidney allograft biopsy. Thus, the 2019 Banff classification retains DSA detection in the serum, histological microvascular inflammation and C4d deposits as the hallmarks of ABMR diagnosis^3^. Not all three criteria are required, as proposed surrogate markers allow several combinations to be accepted (e.g. C4d negative ABMR may be diagnosed with a significant microvascular inflammation in addition to DSA detection, and ABMR without detectable DSA may be diagnosed with microvascular inflammation and C4d deposits).

Nevertheless, the diagnosis of active ABMR remains complex, due to our limited understanding of the full dynamic range of ABMR and the known limitations of the current criteria^4^. Indeed, the morphological scores still lack inter-observer reproducibility, even between experienced nephropathologists^4–6^. A recent study only showed a mild to moderate reproducibility for the glomerulitis and peritubular capillaritis scores, with Cohen’s Kappa of 0.39 and 0.38, respectively^7^. C4d deposits are highly specific to an active antibody-mediated mechanism, but are known to be negative in up to 50% of ABMR cases^5,8^. The DSA criterion has at least two limitations: (i) the heterogeneity among centers in the exhaustivity of their testing and (ii) the growing evidence of the involvement of antibodies targeting non-HLA antigens^9^, which are not easily routinely tested. In addition, a mechanism of microvascular inflammation has recently been described, which is not mediated by antibodies but by NK cells^10^. Finally, validated molecular classifiers have been added as a surrogate marker for an ABMR diagnosis since 2015^11^, although currently they are not widely available and are still struggling to be applied in current global practice.

Treatment of ABMR primarily aims at removing circulating DSA, blocking their effects and/or reducing their production. Glucocorticoids, plasma exchange and intravenous immunoglobulins remain the basis of current therapy^12^. Because this treatment is complex, burdensome and sometimes associated with complications, such as infection and thrombosis, optimizing the diagnostic performance of ABMR by pathologists is a major and primary need.

**Figure 1:**
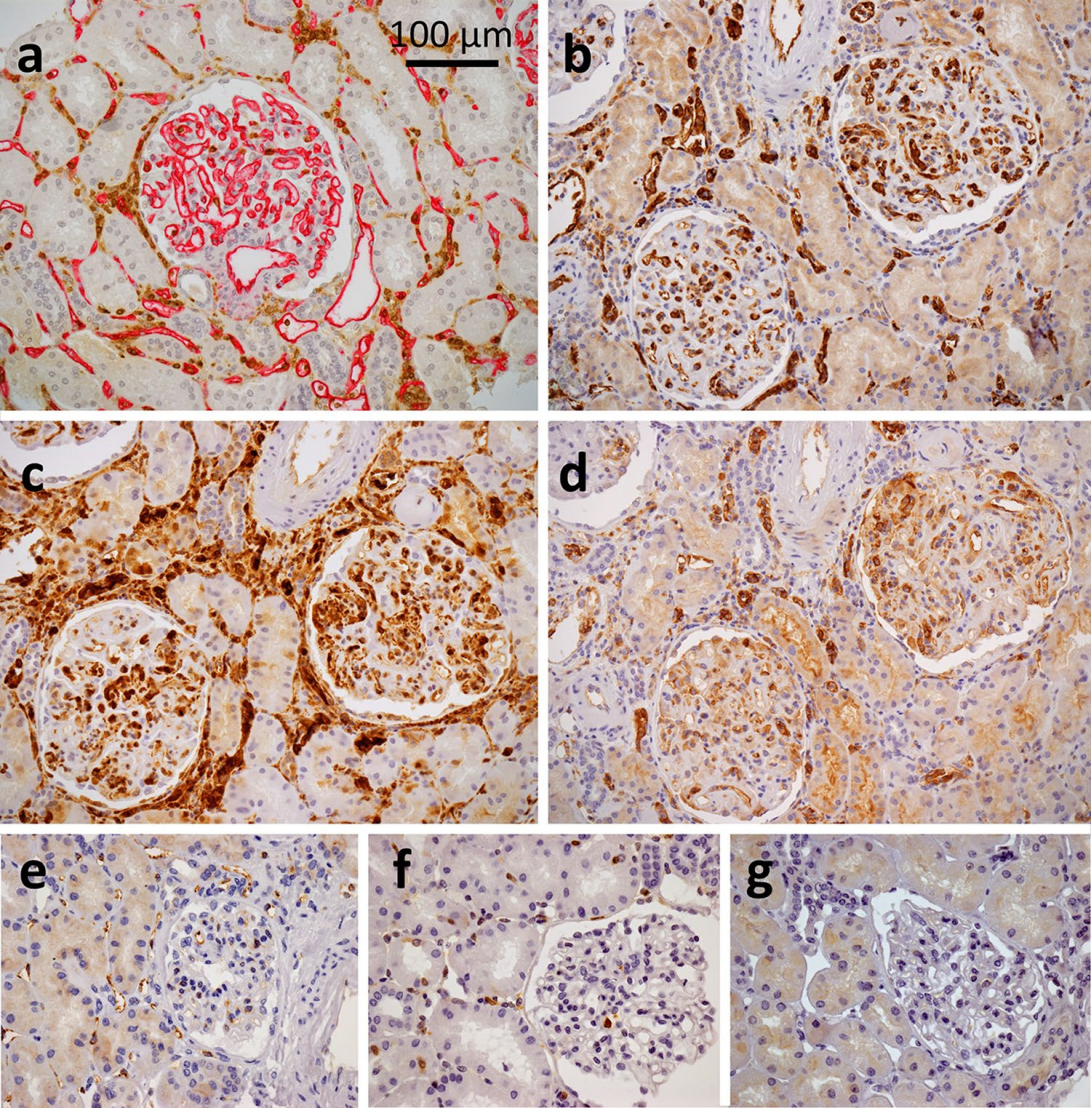
Description of a microcirculation staining pattern with a CD34/CORO1A control case (**a**) and illustrative examples seen in ABMR as compared to a stable graft case with WARS1 (**b**, **e**), TYMP (**c**, **f**) and GBP1 (**d**, **g**), original magnification × 200 (**a**–**d**) and × 400 (**e**–**g**). (**a**) The CD34 highlights endothelial cells of glomeruli and peritubular capillaries, *i.e.* of the microcirculation, in magenta, while CORO1A acts as a pan-leukocyte marker and highlights inflammatory cells in brown, both in the microcirculation and the interstitium. Note the similarity of the expression pattern with a strong and diffuse microcirculation staining, and to a lesser extent in inflammatory cells, obtained with WARS1 (**b**) and GBP1 (**d**). TYMP (**c**) mainly shows a diffuse and strong staining of inflammatory cells, both in the microcirculation and the interstitium, but also a moderate staining of endothelial cells. (**e**): A mild but diffuse staining of endothelial cells is observed in a stable graft case with WARS1. (**f**): In a stable case, TYMP is strongly expressed on a few inflammatory cells, wherever they are, without significant endothelial staining. (**g**): No significant staining of GBP1 in a stable graft case. Abbreviations: ABMR, antibody-mediated rejection; WARS1, tryptophan–tRNA ligase, cytoplasmic; TYMP, thymidine phosphorylase; GBP1, guanylate-binding protein 1.

In a previous study, we analyzed the glomerular proteome modifications during active ABMR compared to stable grafts, using laser microdissection combined with tandem mass spectrometry^13^. We described 77 dysregulated proteins in glomerulitis and highlighted 3 interferon-related proteins, which displayed an overexpression by immunohistochemistry in glomerular endothelial cells during ABMR: WARS1, TYMP and GBP1. Proteomics results suggested their robustness with respect to chronicity and C4d status. Furthermore, through an exploratory approach, we noticed that WARS1, TYMP and GBP1 displayed a microcirculation staining pattern by immunohistochemistry in ABMR cases (Fig. 1), highlighting not only inflammatory but also endothelial cells in both glomeruli and peritubular capillaries.

In the last decade, deep learning-based computer vision surged as one of the best opportunities for more quantitative and reproducible histopathologic evaluations, as well as reordering pathologists’ priorities, by reducing time-consuming and delegable tasks to algorithms. In oncology, deep learning-based approaches have been described not only for diagnostic and prognostic applications^14–16^, but also for prediction of molecular alterations^15,17,18^. In the area of kidney transplantation, recent studies have highlighted the value of deep learning for the identification of abnormal (*i.e.* lesional) allograft biopsies from morphological slides^19^, the prediction of early and long-term graft survival based on baseline and 12-month post-transplant biopsies^20^, the assessment of the C4d staining^21,22^ and the quantitative evaluation of tubulo-interstitial inflammation^23^.

Herein we performed an immunohistochemical analysis of WARS1, TYMP and GBP1 in a selected cohort of kidney allograft biopsies including common graft injuries encountered in routine practice. The aims of the study were to (i) assess the potential value of the microcirculation pattern in the diagnosis of ABMR, as interpreted by four nephropathologists, (ii) evaluate their suitability for a deep learning-based interpretation and classification and (iii) describe the overall expression pattern of WARS1, TYMP and GBP1 in kidney transplantation by immunohistochemistry.

## Materials and methods

### Selection of the cohort

This is a single-center, retrospective, descriptive study analyzing selected kidney allograft biopsies. All cases consisted of renal allograft biopsies, formalin-fixed and paraffin embedded, already performed for diagnosis purposes from August 2011 to February 2016 at the Bordeaux University Hospital. Diagnoses were in accordance with the 2017 Banff classification. Chronic ABMR was defined by light microscopy (≥ cg1b). With the exception of recurrent glomerulopathies, immunofluorescence study with antibodies targeting IgA, IgG, IgM, C3, Kappa and Lambda was negative for all cases. C4d status was assessed by immunofluorescence. As required by the local institution’s ethics board, patients for whom a renal biopsy was eligible were contacted and had the legal time of one month to express their opposition. The study was conducted according to the guidelines of the Declaration of Helsinki, and our clinical database had a French CNIL (Commission Nationale Informatique & Libertés) final agreement, decision 2009-413, n° 1357154, 2 July 2009. No tissues were procured from prisoners.

### Immunohistochemical study and analysis

For immunohistochemistry, 2.5 μm thick sections were performed, dewaxed and rehydrated. Antigen retrieval was performed in a 1 mM Tris–EDTA pH = 9 solution. All staining procedures were performed in an automated autostainer (Dako-Agilent, Santa Clara, United States) using standard reagents provided by the manufacturer. Three commercial primary antibodies were used from the manufacturer Abcam, targeting thymidine phosphorylase (TYMP, mouse, clone P-GF.44C, dilution 1:200), tryptophan–tRNA ligase, cytoplasmic (WARS1, rabbit, clone EPR3423, 1:3000) and guanylate-binding protein 1 (GBP1, mouse, clone OTI1B2, 1:50). The sections were incubated with the corresponding antibody for 45 min at room temperature. EnVision Flex/horseradish peroxidase (Dako-Agilent) was used for signal amplification, revealed by 3,3’-diamino-benzidine (Dako-Agilent). The slides were counterstained with hematoxylin, dehydrated and mounted. Each immunohistochemical assay contained a negative (buffer, no primary antibody) and positive control (transplant nephrectomy with chronic active ABMR lesions). Slides were interpreted by four nephropathologists (B.C., A.V., M.R. and JP.DVH.). They were unaware of the diagnosis and should assess each case as either positive or negative for an active ABMR diagnosis, based on the recognition of a microcirculation staining pattern (Fig. 1).

Specifically, for the CD34/CORO1A double staining, the ImmPRESS Duet Double Staining Polymer Kit was used (MP-7714, Vector Laboratories, Burlingame, United States), with the CD34 antibody (Leica-Novocastra, mouse, QBEnd/10, dilution 1:100) and the coronin-1A antibody (CORO1A/TACO, Abcam, rabbit, EPR19467-36, 1:3000).

### Deep learning analysis for classification from virtual slides

**Figure 2:**
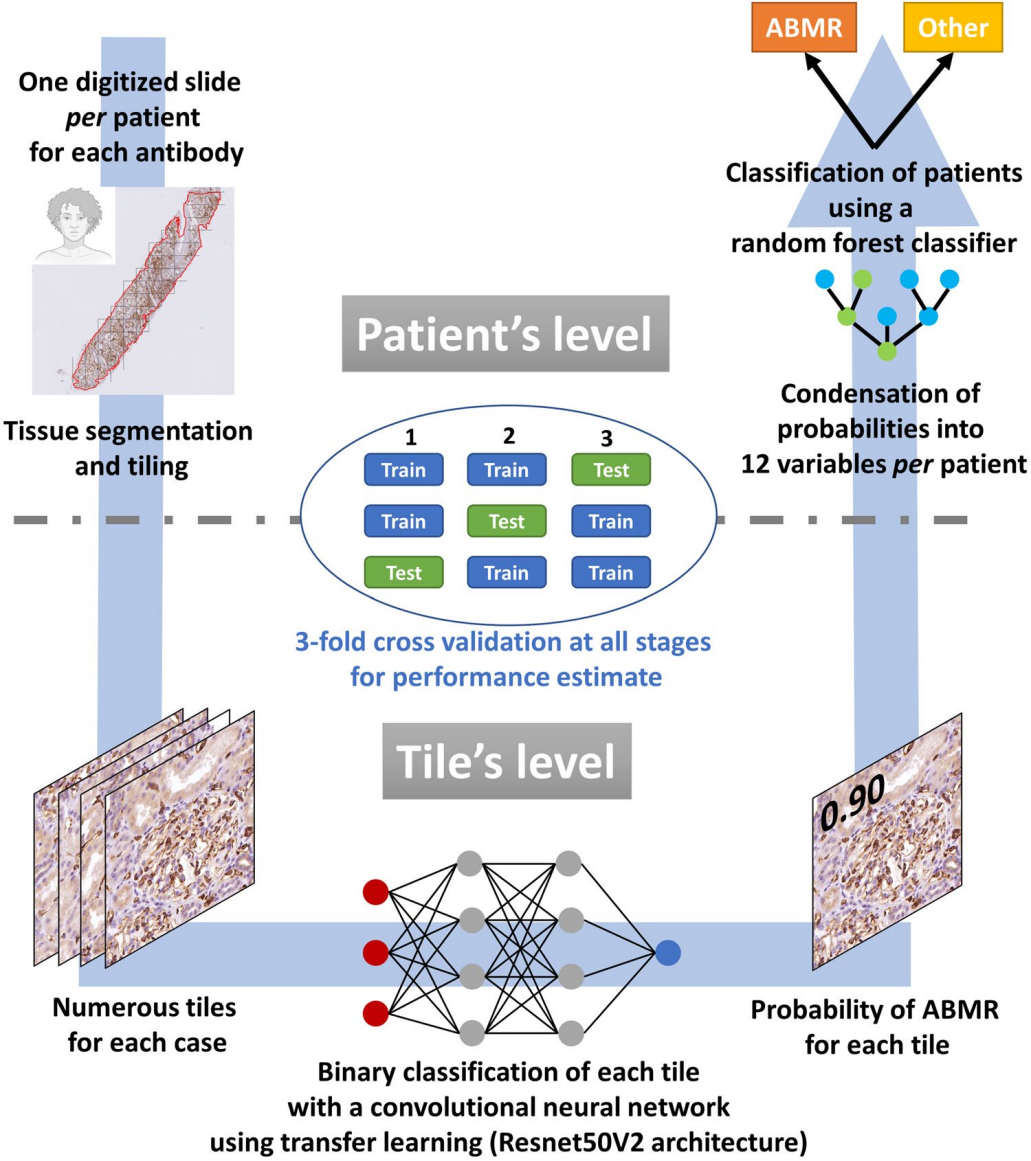
Overall deep learning-based analytical strategy of the study. A deep learning approach was used to build models for the sequential binary classification ABMR *versus* Other diagnosis for each antibody. Briefly, each whole slide image, one *per* patient and *per* antibody, was cropped in square tiles and, from these, two models were trained for a sequential binary classification. Firstly, a convolutional neural network, namely the pre-trained Resnet50V2 architecture, was trained at the tile level and secondly a random forest classifier was trained at the patient level (*i.e.* whole slide image), based on the output of model 1 for all tiles of a considered patient. Internal validation was performed for the evaluation of models’ performance, using a threefold cross-validation and by maintaining data split for both the training of models 1 and 2. Abbreviation: ABMR, antibody-mediated rejection. Created with BioRender.com.

Deep learning for computer vision was used to train models for the binary classification ABMR/Other diagnosis for each antibody. The overall analytical strategy is illustrated in Fig. 2. All analyzed slides were anonymized and digitized into the ndpi format using a Hamamatsu NANOZOOMER 2.0HT at the objective × 20 (resolution 0.46 μm/pixel). Using the QuPath 0.2.3 software^24^, the renal parenchyma was manually annotated for each slide, defining the regions of interest. Each region of interest was then segmented into square tiles of 512 × 512 pixels. Tiles were numbered for each case and exported into the jpeg format, according to the Aachen protocol for Deep Learning Histopathology^25^.

Deep learning models were trained using the keras library with the TensorFlow backend^26,27^. As illustrated in Fig. 2, two models were trained for a sequential binary classification ABMR *versus* Other diagnosis. Firstly, a convolutional neural network (CNN, model 1) was trained at the tile level and secondly a random forest classifier (model 2) was trained at the patient level (i.e. whole slide image), based on the output of model 1 for all tiles of the considered patient. Internal validation was carried out to estimate models’ performance, using a threefold cross-validation. To ensure a balanced split of both tiles and patients throughout both models 1 and 2, stratified folds were created using the StratifiedGroupKFold function of the scikit-learn library. Five repeated cross-validation procedures were conducted to estimate the overall performance variability of the whole classification. Due to the small amount of available data and the lack of a holdout set, hyperparameter tuning was reduced to a minimum, to allow an honest estimate of overall performance. Hyperparameter tuning was empirically performed from the first fold of the first iteration of the cross-validation with the WARS1 antibody and hyperparameters were then identically set for all neural network models of the cross-validation and all antibodies without further tuning. The area under the receiver operating characteristic curve (AUC) was set as performance metric.

Transfer learning was performed for the training of model 1, using the pre-trained network Resnet50V2, available in the keras library. The Resnet50V2 model was loaded with the trained weights based on the ImageNet dataset, excluding the final classification layer. Instead, a GlobalAveragePooling and a Dense layer of 1 neuron were added, the latter with a sigmoid activation function. To make the model more generalizable, i.e. more robust against image variations, we applied image data augmentation during training. As such random image alterations were performed such as horizontal and vertical flips, rotation and channel shifts. To account for the imbalance of the dataset between ABMR and Other diagnosis cases, we used a weighted binary cross-entropy loss function. Thus, a misclassification in the underrepresented ABMR class gave a higher error than the majority “Other diagnosis” class. The model 1 was firstly trained for one epoch with all convolutional layers kept frozen (weights non-trainable), with an Adam optimizer, a learning rate of 1e-04 and a batch size of 64. Secondly, the last two convolutional blocks (blocks 4 and 5) were unfrozen for fine-tuning and trained for 10 epochs, with a learning rate of 1e-05. The weights of the epoch achieving the best AUC in the validation set were restored.

Because each patient had a different number of tiles, 12 variables were created based on the output of model 1 for all tiles of a considered patient. Seven variables were defined by simple descriptive statistics: mean of all tiles’ predictions, median, minimum and maximum values, standard deviation, quantiles 25 and 75. In addition, to better assess the consistency of in situ expression, we added 5 variables reflecting the consistency of local expression pattern. For this, we performed a one-dimensional average pooling from all ordered tiles of each patient, with a pool size of 10 and a stride of 5. The 5 variables were: minimum and maximum values, quantiles 25 and 75 and standard deviation. Overall, these 12 variables enabled an input for model 2 of the same shape for each patient, regardless of the number of tiles, compatible with most machine learning methods. Model 2 consisted of a random forest classifier, build with the scikit-learn library, with 100 trees, trained while maintaining the train/validation split for all three folds of the cross-validation. For each trained model 2, 50 iterations were performed and the mean of the validation AUC was retained. Similarly, best threshold was computed (closest top-left method) and corresponding sensitivity and specificity were averaged. Performance for each cross-validation iteration were then averaged, with weighted means calculated for sensitivity and specificity respectively based on the proportion of ABMR and Other cases from each fold. Finally, performance from the 5 repeated cross-validation procedures were averaged.

To allow for a visual explanation of the CNN classification, we used the Gradient-weighted Class Activation Mapping (Grad-CAM) approach^28^. Briefly, this method uses the final convolutional layer of a trained model to produce a localization map (heatmap), highlighting important regions in the image for class consideration. The heatmap is then superimposed onto the original image. Because Resnet50V2 is not directly suited for this implementation, we used the Xception architecture instead, which we trained in a similar manner as Resnet50V2. The last convolutional layer was set to the “block14_sepconv2_act” layer.

### Software and statistical analysis

All deep learning models were trained using the keras library with the TensorFlow backend^26,27^, using either a Tesla T4 or a P100 as graphics processing units. Area under the curves were calculated using the scikit-learn library. All other statistical analyses were performed using the R software, version 4.1.1^29^. Cohen’s kappa was computed for the evaluation of inter-observer reproducibility between two pathologists, and Light’s kappa for the overall inter-observer reproducibility between all four pathologists. Plots were performed using the ggplot2 package, version 3.3.5, and correlation analyzes with the cor.test function. In order to easily compare pathologists and deep learning interpretations of immunomarkers, a majority rule was applied to pathological interpretations, where each case was classified according to the report of most pathologists. In case of ties, the interpretation of the pathologist B.C. was retained.

## Results

### Main clinical, biological and histological characteristics of included patients

Overall, 54 patients with corresponding kidney allograft biopsy were retrospectively included in this study, including 17 with an active ABMR diagnosis and 37 differential diagnoses commonly encountered in kidney transplantation. From the 17 active ABMR cases, five were C4d positive by immunofluorescence and seven displayed chronic antibody-mediated glomerular injuries (≥ cg1b). All ABMR cases had anti-HLA DSA in their serum, with a median [IQR] mean fluorescence intensity of the immunodominant DSA of 4715 [2500–7500]. Eleven of 17 patients had de novo DSA. The 37 cases of differential diagnoses consisted of: T cell-mediated rejections (n = 6), infections (3 polyomavirus nephropathies and 2 acute pyelonephritides), acute tubular injuries (n = 5), recurrent or de novo glomerular nephropathies (3 IgA nephropathies and 2 membranous nephropathies), non-humoral thrombotic microangiopathies (n = 5), isolated C4d positivity (n = 3), chronic ABMR without activity (g0 ptc0, n = 5) and stable graft cases in ABO incompatible transplantation (one year protocol biopsies without acute lesion, n = 3). Main clinical, biological and histological characteristics are displayed in Table 1.

**Table 1:** Main clinical, biological and histological characteristics of the cohort.

	Active ABMR		Other diagnosis	
	*n*		*n*	
**Recipient**	17		37	
Age at the time of biopsy, year, median [IQR]		52 [48–63]		47 [32–64]
Male, *n* (%)		10 (60)		22 (60)
ESRD causes, *n* (%)				
Glomerulonephritis		3 (18)		9 (24)
Diabetes		1 (6)		4 (11)
Hereditary		5 (29)		11 (30)
Tubulo-interstitial disease		0 (0)		0 (0)
Vascular nephropathy		0 (0)		3 (8)
Uropathy		4 (23.5)		8 (22)
Unknown		4 (23.5)		2 (5)
**Donor**				
Age, median [IQR]	17	57 [41–71]	36	56 [49–62]
Male, *n* (%)	16	11 (69)	35	22 (63)
Living donor, *n* (%)	17	1 (6)	36	10 (28)
**Anti-HLA antibodies at the time of transplantation**	12		33	
No evidence of anti-HLA antibodies, *n* (%)		4 (33)		23 (70)
Evidence of anti-HLA antibodies but not DSA, *n* (%)		2 (17)		3 (9)
DSA, *n* (%)		6 (50)		7 (21)
**DSA at the time of the ABMR diagnosis**	17		37	
DSA, *n* (%)		17 (100)		6 (16)
Class I DSA, *n* (%)		8 (47)		3 (8)
Class II DSA, *n* (%)		13 (76)		4 (11)
De novo DSA, *n* (%)		11 (65)		3 (8)
Immunodominant DSA MFI, median [IQR]		4715 [2500–7500]		0 [0–0]
**Clinical and biological parameters at the time of biopsy**	17		37	
Months post-transplantation of biopsy, median [IQR]		23.8 [8.0–70.4]		8.6 [2.8–48.1]
Biopsy indication, for cause, *n* (%)		15 (88)		30 (81)
eGFR at diagnosis, ml/min/1.73 m^2^, median [IQR]		34 [25–43]		37 [24–51]
Urine protein/creatinine ratio at diagnosis, mg/mmol, median [IQR]		89.6 [12.5–200.5]		99.7 [28.0–185.9]
**Histological parameters – Banff scoring**	17		37	
Total number of glomeruli, median [IQR]		10 [9–13]		13 [10–16]
Number of globally sclerotic glomeruli, median [IQR]		1 [0–2]		1 [0–2]
Banff scoring				
Glomerulitis score				
g > 0, *n* (%)		12 (71)		0 (0)
g, mean (SD)		1.1 (1.0)		0.0 (0.0)
Peritubular capillaritis				
ptc > 0, *n* (%)		17 (100)		3 (8)
ptc, mean (SD)		1.8 (0.4)		0.1 (0.4)
Microvascular inflammation				
g + ptc ≥ 2, *n* (%)		17 (100)		1 (3)
Glomerular basement membrane double contours				
≥ cg1b, *n* (%)		7 (41)		9 (24)
cg, mean (SD)		0.6 (0.8)		0.5 (0.9)
Mesangial matrix expansion				
mm > 0, *n* (%)		5 (29)		8 (22)
mm, mean (SD)		0.4 (0.7)		0.4 (0.8)
C4d score				
C4d > 0, *n* (%)		5 (29)		7 (19)
C4d, mean (SD)		0.9 (1.4)		0.5 (1.1)
Interstitial inflammation in unscarred cortex				
i > 0, *n* (%)		12 (71)		15 (41)
i, mean (SD)		1.1 (1.0)		0.7 (0.9)
Tubulitis in unscarred cortex				
t > 0, *n* (%)		10 (59)		11 (30)
t, mean (SD)		0.9 (1.0)		0.7 (1.1)
Intimal arteritis				
v > 0, *n* (%)		0 (0.0)		0 (0)
v, mean (SD)		0.0 (0.0)		0.0 (0.0)
Total inflammation				
ti > 0, *n* (%)		15 (88)		21 (57)
ti, mean (SD)		1.5 (0.8)		1.0 (1.0)
Interstitial inflammation in scarred cortex				
i-IFTA > 0, *n* (%)		14 (82)		33 (89)
i-IFTA, mean (SD)		1.9 (1.2)		1.9 (1.1)
Tubulitis in scarred cortex				
t-IFTA > 0, *n* (%)		13 (76)		20 (54)
t-IFTA, mean (SD)		1.2 (0.9)		0.9 (1.0)
Interstitial fibrosis				
ci > 0, *n* (%)		15 (88)		36 (97)
ci, mean (SD)		1.5 (0.9)		1.6 (0.8)
Tubular atrophy				
ct > 0, *n* (%)		15 (88)		36 (97)
ct, mean (SD)		1.5 (0.9)		1.6 (0.8)
Arteriolar hyalinosis				
ah > 0, *n* (%)		9 (53)		7 (19)
ah, mean (SD)		0.8 (1.0)		0.4 (0.8)
Vascular fibrous intimal thickening				
cv > 0, *n* (%)		8 (47)		10 (27)
cv, mean (SD)		0.9 (1.1)		0.4 (0.8)

*ABMR* Antibody-mediated rejection, *IQR* Interquartile range, *ESRD* End-stage renal disease, *HLA* Human leukocyte antigens, *DSA* Donor-specific antibodies, *eGFR* Estimated glomerular filtration rate, *MFI* Mean fluorescence intensity.

### Performance of pathologists for ABMR diagnosis with each antibody

All slides were interpreted by four nephropathologists (B.C., A.V., M.R. and JP.DVH.). They were unaware of the diagnosis and should assess each case as positive or negative for an active ABMR diagnosis based on the recognition of a microcirculation staining pattern. The microcirculation staining was defined as a positive staining of one or both microcirculation compartment (i.e. glomerular and/or peritubular capillaries), with a diffuse pattern for WARS1 and TYMP, while a focal pattern was considered for GBP1 (Fig. 1). This definition was based on the initial study that revealed these proteins by mass spectrometry, including 21 ABMR and 8 stable graft cases, which was used as a training cohort for the pathologists^13^.

Performance of the pathologists are summarized in Tables 2 and 3 (see also Supplemental Tables S1–S4 for more details). Overall, TYMP had the best diagnostic performance in this cohort, with a mean sensitivity (Se) of 88% (± 9), mean specificity (Sp) of 86% (± 5), and a substantial agreement (Light’s κ = 0.73). WARS was slightly less sensitive and specific (mean Se = 80% ± 11, Sp = 81% ± 5) but also with a substantial agreement (κ = 0.64). Finally, GBP1 had the lowest sensitivity (mean Se = 60% ± 6), with a mean specificity of 90% ± 3 and a substantial interobserver reliability (κ = 0.68). While applying a majority rule for pathologists’ interpretation, 11 of 12 cases of C4d negative ABMR were properly identified with TYMP, 7 of 11 with WARS1 and 6 of 11 with GBP1 (Supplemental Table S5). There was no significant association between immunostain positivity and the C4d status: *p* = 0.24 for WARS1, *p* = 0.45 for TYMP and *p* = 0.57 for GBP1 (Fisher’s exact test, Table 3). Of note, there was no obvious morphological difference of staining in active ABMR cases depending on the C4d status. As displayed in Table 3, false positives were mainly due to (i) some infection and T cell-mediated rejection (TCMR) cases, where a marked and diffuse interstitial inflammation led to a misleading endothelial positivity on peritubular capillaries or (ii) chronic antibody-mediated rejection cases thought to be non-active according to the Banff classification, i.e. without microvascular inflammation, g0 ptc0 (see also Supplemental Figs. S1 and S2). False negatives were mainly due to staining judged as too focal and/or too weak.

**Table 2:** Overall performance of the pathologists and of a deep learning-based classification approach in the diagnosis of ABMR with the WARS1, TYMP and GBP1 antibodies by immunohistochemistry.

	WARS1				TYMP				GBP1			
	Se (%)	Sp (%)	Kappa	AUC, mean (SD)	Se (%)	Sp (%)	Kappa	AUC, mean (SD)	Se (%)	Sp (%)	Kappa	AUC, mean (SD)
P1	75	86			88	86			60	86		
P2	69	84			75	89			67	89		
P3	94	76			94	78			53	92		
P4	81	78			94	89			60	92		
Mean (SD), P1 to P4	80 (11)	81 (5)	0.64		88 (9)	86 (5)	0.73		60 (6)	90 (3)	0.68	
Deep learning	84 (4)	92 (2)		0.89 (0.02)	77 (5)	84 (3)		0.80 (0.04)	88 (6)	86 (6)		0.89 (0.04)

Slides were interpreted by four nephropathologists (B.C., A.V., M.R. and JP.DVH.). They were unaware of the diagnosis and should assess each case as positive or negative for an active ABMR diagnosis based on the recognition of a microcirculation pattern of staining. For each antibody, pathologists were trained using the immunostains obtained from the initial study that revealed these proteins by mass spectrometry^13^. Light’s Kappa are provided for estimation of inter-observer reliability. A deep learning approach was used to build models for the binary classification ABMR *versus* Other diagnosis for each antibody. Two models were trained for a sequential binary classification. Firstly, a convolutional neural network (Resnet50V2) was trained at the tile level and secondly a random forest classifier was trained at the patient level (i.e. whole slide image), based on the output of model 1 for all tiles of a considered patient. Internal validation was performed for the evaluation of models’ performance, using 5 iterations of a threefold cross-validation. Average results of the models’ performance on the validation set are displayed.

*ABMR* Antibody-mediated rejection, *WARS1* Tryptophan–tRNA ligase, cytoplasmic, *TYMP* Thymidine phosphorylase, *GBP1* Guanylate-binding protein 1, *Se* Sensitivity, *Sp* Specificity, *AUC* Area under the receiver operating characteristic curve, *P* Pathologist, *SD* Standard deviation.

**Table 3:** Interpretation results of WARS1, TYMP and GBP1 antibodies by immunohistochemistry for predicting active ABMR by pathologists and deep learning.

Diagnosis	WARS1 positivity for active ABMR			TYMP positivity for active ABMR			GBP1 positivity for active ABMR		
	Number of total cases (n = 53)	Number of cases considered positive, n (%)		Number of total cases (n = 52)	Number of cases considered positive, n (%)		Number of total cases (n = 52)	Number of cases considered positive, n (%)	
		Pathologists	Deep learning		Pathologists	Deep learning		Pathologists	Deep learning
Active ABMR	16	12 (75)	13 (81)	16	14 (88)	13 (81)	15	7 (47)	14 (93)
Other diagnosis	37	5 (14)	2 (5)	36	3 (8)	4 (11)	37	3 (8)	5 (14)
**Including**									
Active ABMR, C4d positive	5	5 (100)	5 (100)	4	3 (75)	3 (75)	4	1 (25)	4 (100)
Active ABMR, C4d negative	11	7 (64)	8 (73)	12	11 (92)	10 (83)	11	6 (55)	10 (91)
Non-active cABMR	5	2 (40)	1 (20)	5	2 (40)	1 (20)	5	1 (20)	1 (20)
Isolated C4d	3	1 (33)	0 (0)	3	1 (33)	1 (33)	3	1 (33)	0 (0)
SG ABO incompatible	3	0 (0)	0 (0)	3	0 (0)	0 (0)	3	0 (0)	0 (0)
Non-humoral TMA	5	0 (0)	0 (0)	5	0 (0)	0 (0)	5	0 (0)	0 (0)
Acute TCMR	6	1 (17)	1 (17)	6	0 (0)	1 (17)	6	0 (0)	3 (50)
Infection (PVN, APN)	5	1 (20)	0 (0)	4	0 (0)	1 (25)	5	1 (20)	1 (20)
ATI	5	0 (0)	0 (0)	5	0 (0)	0 (0)	5	0 (0)	0 (0)
Recurrent GN	5	0 (0)	0 (0)	5	0 (0)	0 (0)	5	0 (0)	0 (0)

To easily compare pathologists and deep learning interpretations of the immunomarkers, a majority rule was applied on pathological interpretations, where each case was classified according to the report of most pathologists. In case of ties, the interpretation of the pathologist B.C. was retained. As for deep learning analysis, the three folds of one iteration of the cross-validation was used to classify samples. Please note that this iteration performance is logically slightly different than the average performance displayed in Table 3. Variations in total number of cases are due to insufficient remaining material for interpretation.

*ABMR* Antibody-mediated rejection, *WARS1* Tryptophan–tRNA ligase, cytoplasmic, *TYMP* Thymidine phosphorylase, *GBP1* Guanylate-binding protein 1, *cABMR* Chronic antibody-mediated rejection, *SG* Stable graft, *TMA* Thrombotic microangiopathy, *TCMR* T cell-mediated rejection, *PVN* Polyomavirus nephropathy, *APN* Acute pyelonephritis, *ATI* Acute tubular injuries, *GN* Glomerulonephritis.

### Deep learning-based classification, visual interpretation and correlation with the Banff scores

We assessed the suitability of deep learning for the diagnosis of ABMR with the immunomarkers WARS1, TYMP and GBP1. The overall analytical strategy is illustrated in Fig. 2. Briefly, we used a convolutional neural network (CNN)-based pipeline for the binary classification of the immunostains as ABMR or other diagnosis, analyzed after cropping whole slide images into multiple square tiles. Internal validation was performed to assess model performance using 5 iterations of a threefold cross-validation (see also Supplemental Table S6 for an exhaustive description of the process of model performance evaluation). Table 2 displays the performance of each antibody for an ABMR diagnosis. For WARS1, the mean (± standard deviation) area under the curve (AUC) in the validation sets was 0.89 (± 0.02), with a mean sensitivity of 84% (± 4) and specificity of 92% (± 2). For TYMP, mean AUC was 0.80 (± 0.04), with mean Se = 77% (± 5) and Sp = 84% (± 3). As for GBP1, mean AUC was 0.89 (± 0.04), with mean Se = 88% (± 6) and Sp = 86% (± 6). Like pathologists, false positives mainly concerned some TCMR and non-active chronic ABMR cases (Table 3). Indeed, the comparison of pathologists’ interpretation (majority rule) and the deep learning approach showed a substantial agreement for WARS1 (κ = 0.73, *p* = 8.8E−08) and an almost perfect agreement for TYMP (κ = 0.83, *p* = 2.7E−09). However, only a fair agreement was seen for GBP1 (κ = 0.31, *p* = 0.01), where the deep learning strategy had a remarkably better sensitivity than pathologists, but displayed less specificity (especially 3 false positives of TCMR cases).

**Figure 3:**
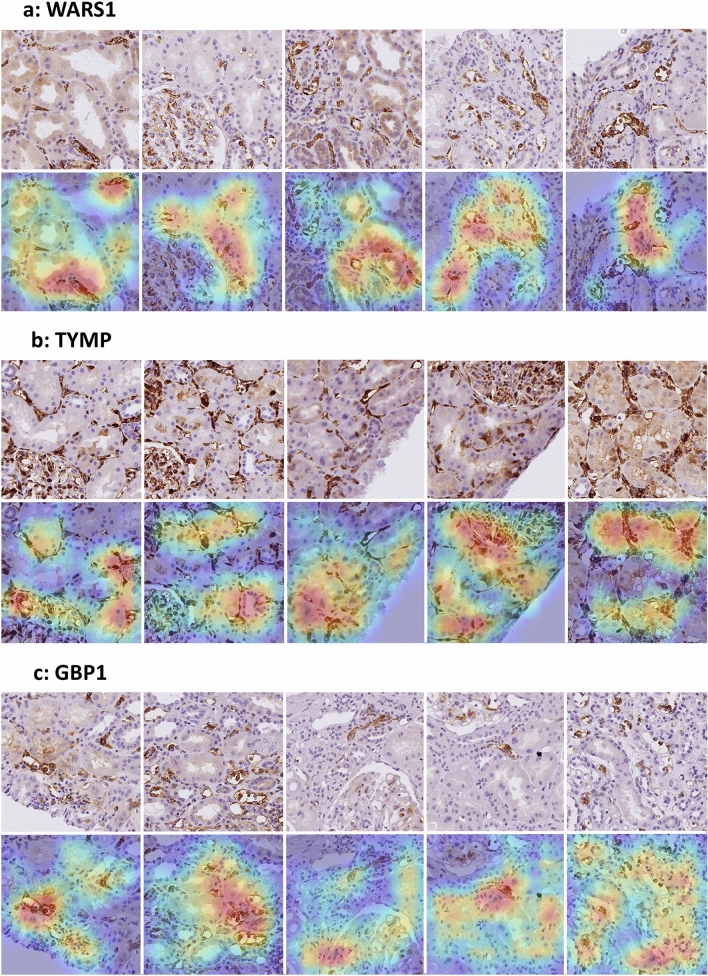
Illustrative morphological patterns of (**a**) WARS1, (**b**) TYMP and (**c**) GBP1 associated by the convolutional neural network with the active ABMR class, using the Grad-CAM approach. For each antibody, 5 tiles (512 × 512 pixels) among the most associated with the ABMR class are displayed, with the corresponding heatmap presented below (from blue to red, where red represents regions of utmost importance by the neural network for class consideration). Notice that, for each antibody, the neural network focused on capillaries lined by moderately to strongly stained endothelial cells, sometimes associated with inflammatory cells, and particularly considering peritubular capillaries, rarely glomerular capillaries. Endothelial staining is diffuse with WARS1 and TYMP, focal with GBP1.

We then used the Gradient-weighted Class Activation Mapping (Grad-CAM) approach^28^, to allow a visual interpretation of the deep learning approach, by exploring important regions used by the CNN for image classification. Figure 3 shows illustrative examples of native tiles and corresponding heatmaps in some of the most confident image classifications as ABMR cases for each antibody (see also Supplemental Fig. S3 for tiles associated with the “Other diagnosis” class). For each antibody, the CNN interpretation supported the microcirculation staining pattern of the ABMR classification, focusing on capillaries lined by moderately to strongly stained endothelial cells, sometimes associated with inflammatory cells. This interpretation was particularly manifest for peritubular capillaries, but rarer for glomerular capillaries.

We then assessed the correlation between the prediction of ABMR from the deep learning framework (i.e. output of model 2) with the histological Banff scores. All three antibodies displayed their best correlation with the microvascular inflammation scores, i.e. the sum g + ptc, with a Spearman’s rho of 0.65 (*p* = 1.8E−07) for WARS1, 0.44 (*p* = 1.0E−03) for TYMP and 0.72 (*p* = 1.9E−09) for GBP1. The following single scores were peritubular capillaritis, with a rho of 0.63 (*p* = 4.2E−07), 0.42 (*p* = 2.0E−03) and 0.69 (*p* = 1.8E−08), and glomerulitis, with a rho of 0.54 (*p* = 3.4E−05), 0.42 (*p* = 1.7E−03) and 0.64 (*p* = 3.1E−07), respectively. Significant correlations were also found, to a lesser extent, with the tubulo-interstitial inflammation scores. No significant correlations were found with the cg score (double contour of the glomerular basement membrane, hallmark of chronic antibody-mediated damages) and the C4d score (see also Supplemental Table S7).

### Overall expression pattern of WARS1, TYMP and GBP1 in kidney allograft biopsies

**Figure 4:**
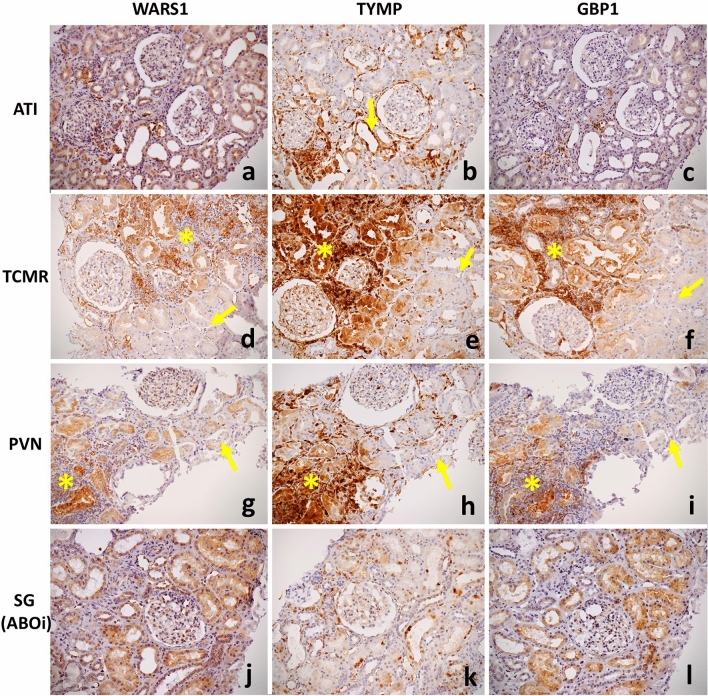
Illustration of the immunostains WARS1, TYMP and GBP1 obtained in the main analyzed differential diagnoses of ABMR, including acute tubular injury (**a**–**c**), T cell-mediated rejection (**d**–**f**), polyomavirus nephropathy (**g**–**i**) and stable graft in ABO-incompatible transplantation (**j**–**l**). Injured tubular cells showed cytoplasmic positivity with variable intensity, with a mild to moderate staining with WARS1 (**a**) and GBP1 (**c**), and more strongly and consistently with TYMP (arrow, b). There was no diffuse endothelial staining in such cases of acute tubular injury (**a**–**c**). In T cell-mediated rejection (**d**–**f**) and polyomavirus nephropathy (**g**–**i**), interstitial infiltrate is moderately to strongly stained, as well as nearby injured tubular cells (asterisks). Endothelial cells of peritubular capillaries were frequently stained in these areas, but exceptionally glomerular endothelial cells. Afar from these areas of tubulo-interstitial inflammation, note the absence of significant endothelial staining of the microcirculation, similarly to constitutive staining (arrows). (**j**–**k**): In this stable graft case, a mild positivity is observed with WARS1 and GBP1 in tubular cells. Some sparse inflammatory cells are strongly stained with TYMP, and a moderate staining is observed in a few tubular cells. No overt and diffuse endothelial staining is observed whatever the antibody. Abbreviations: WARS1, tryptophan–tRNA ligase, cytoplasmic; TYMP, thymidine phosphorylase; GBP1, guanylate-binding protein 1; SG, stable graft; ABOi: ABO-incompatible kidney transplantation; ATI, acute tubular injury; TCMR: T cell-mediated rejection; PVN: polyomavirus nephropathy.

In summary, the overall expression pattern of WARS1, TYMP and GBP1, as observed in this cohort of kidney allograft biopsies, is displayed in Table 4. As illustrated in Figs. 1 and 4, all three antibodies showed a cytoplasmic and, to a certain extent, nuclear positivity. The constitutive staining, as observed in stable graft cases, consisted of a weak and often segmental endothelial cell positivity for WARS1. With TYMP, a few inflammatory cells and atrophic tubules were constitutively positive in such cases, while no specific staining was observed with GBP1. All three antibodies stained inflammatory infiltrates, but with various intensity and pattern (diffuse or focal), whatever the renal compartment (glomeruli, tubules, interstitium or vessels). TYMP showed a diffuse and strong staining in inflammatory cells, while the staining was moderate and more focal with WARS1 and GBP1. Injured tubules (tubulitis and acute tubular injuries) were consistently stained with TYMP, more focal with WARS1 and GBP1. Endothelial staining of peritubular capillaries was observed with all three antibodies in cases of adjacent interstitial infiltrate, but glomerular endothelial cells were usually negative in this setting (Supplemental Fig. S1). As already mentioned, a diffuse endothelial staining, also called microcirculation staining pattern, was mostly found in ABMR. This microcirculation staining could be displayed on one or both microcirculation compartment (i.e. glomerular and/or peritubular capillaries) depending on the cases, and was diffuse for TYMP and WARS and more focal for GBP1.

**Table 4:** Main observed expression patterns with the WARS1, TYMP and GBP1 antibodies in kidney allograft biopsies, with a focus on ABMR condition.

	Glomerular endothelial cells			Endothelial cells of peritubular capillary			Endothelial cells of arteries		Tubular cells				Inflammatory cells
	SG	ABMR	Adjacent Interstitial infiltrate	SG	ABMR	Adjacent Interstitial infiltrate	SG	ABMR	SG	ABMR	ATI	Adjacent Interstitial infiltrate	All conditions
WARS1	+	++/+++	+	+	++/+++	++/+++	+	Variable	0	0	+	+/++	+/++
TYMP	+/−	+ +	+ /−	+/−	++/+++	++/+++	0	Variable	0	0	+ +	++/+++	++/+++
GBP1	0	+/ + + , focal	0	0	+/++, focal	+/++	0	Variable	0	0	+/++	+/++	+/++, scattered

The first line refers to the cell type where the staining is analyzed, while the second line refers to a condition. Interstitial infiltrate can notably refer to T cell-mediated rejection processes as well as infections. Please note that other cell types are sometimes also stained but are not displayed here as such stains appeared less consistent. Indeed, glomerular epithelial cells were sometimes stained with TYMP in ABMR, and also mesangial cells during glomerulitis lesions.

*ABMR* Antibody-mediated rejection, *SG* Stable graft, *ATI* Acute tubular injury, *WARS1* Tryptophan–tRNA ligase, cytoplasmic, *TYMP* Thymidine phosphorylase, *GBP1* Guanylate-binding protein 1.

## Discussion

Antibody-mediated rejection is the leading cause of allograft failure in kidney transplantation and as such is one of the major causes of the lack of improvements in long-term allograft survival. The gold standard of ABMR diagnosis is the morphological examination of an allograft biopsy. Despite several revisions to the Banff classification, ABMR diagnosis remains challenging, with limited inter-observer reproducibility and efficient immunomarker availability. While overestimating rejection can lead to excessive treatment, unnecessary follow-up biopsies and exacerbate the burden of patient anxiety, underestimation can lead to treatment delays and ultimately a worse graft outcome. In this context, highlighting immunomarkers of microvascular inflammation, combined with a deep learning framework, could help to mitigate these challenges. Herein we described for the first time, to our knowledge, the expression pattern of WARS1, TYMP and GBP1 by immunohistochemistry in kidney transplantation, and showed that they distinctively highlight the microcirculation during ABMR, as identified by both the pathologists and a deep learning framework, with promising diagnostic value.

TYMP, WARS1 and GBP1 are IFNγ-induced proteins, which we found enriched by mass spectrometry at a protein level in glomeruli with antibody-mediated injuries, as compared to stable graft controls^13^. WARS1 and GBP1 are also among the most relevant rejection transcripts described by whole-biopsy microarray analysis in kidney transplantation. They are especially described as universal rejection-associated transcripts (both ABMR and T cell-mediated rejection, TCMR), enhanced in parenchymal, endothelial cells and macrophages^30^. Moreover, recent studies using single-cell RNA sequencing strategies in kidney rejection highlighted that WARS1 transcript was particularly upregulated during ABMR in monocytes, especially the CD16a + subpopulation^31^, and in endothelial cells and cycling cells^32^. Wu et al*.* also showed, in a case of mixed rejection, an upregulation of GBP1 in endothelial cells, monocytes, cycling cells and some epithelial cells, especially from the proximal tubule. As for TYMP, an upregulation was seen in monocytes, B cells, cycling cells and to a lesser extent in the proximal tubule^32^.

Indeed, we found an overexpression of these 3 proteins by immunohistochemistry in several cell-types at a protein level during ABMR: inflammatory cells, but also injured cells like tubular cells, especially with TYMP during acute tubular injuries or tubulitis-related injuries (infections and TCMR). Endothelial cells of peritubular capillaries showed an overexpression of WARS1, TYMP and GBP1 in context of nearby interstitial inflammatory infiltrate, overexpression which was rarely found in glomerular endothelial cells in this setting. More importantly, endothelial cells from one or both microcirculation compartments (glomeruli and/or peritubular capillaries) showed an overexpression of these 3 proteins in cases with ABMR features. We defined this as a microcirculation staining pattern. This finding appears relevant from a pathophysiological point of view, as ABMR is in essence a disease of endothelial cells targeted by circulating DSA. Moreover, our deep learning-based approach represented an unbiased approach of markers interpretation and further supported the microcirculation pattern in active ABMR. Indeed, the CNN focused on microvascular structures for its decision process, especially on peritubular capillaries. The relative rarity of glomerular sections (about 13% of the tiles contained a glomerulus), explained in part that the tiles most associated with the ABMR class did not frequently contain a glomerulus.

In this study, WARS1 and TYMP were the most suitable antibodies for pathologist interpretation in the diagnosis of ABMR, achieving reasonable performance with a substantial inter-observer reliability. In addition, we have produced a “proof-of-concept” of the usefulness of a deep learning strategy with microcirculation immunomarkers in ABMR diagnosis, with performance of a similar magnitude than pathologists. This finding is of importance, as deep learning can theoretically suppress inter-observer variability of interpretation, one of the greatest burdens on pathologists. A recent large-scale study of kidney transplant biopsies showed good performance of deep learning from morphological slides for the classification normal/disease biopsies (mean AUC of 0.83). However, lower performing results were seen for the distinction universal rejection *versus* other transplant injuries (mean AUC of 0.61)^19^. The addition of the immunomarkers WARS1, TYMP and/or GBP1 could be of interest in this context.

Although all active ABMR cases had anti-HLA DSA in this study, WARS1 and TYMP showed a diffuse microcirculation pattern in most C4d negative cases, highlighting their potential interest in this context. As non-anti-HLA DSA are still not routinely tested, the diagnosis of such ABMR cases with no detectable anti-HLA DSA ultimately relies on C4d in daily practice, which is not known to perform well in this setting^33,34^, with up to 86% of C4d-negative cases in a recent transcriptomic study^35^. By highlighting a diffuse endothelial staining by immunohistochemistry, WARS1 and TYMP could be of great interest in these cases and this needs to be assessed in further studies. Moreover, WARS1 and TYMP negativity in ABO-incompatible subnormal biopsies is a promising result, in these patients where C4d deposits are constitutively present and thus nonspecific to an active antibody-mediated process.

In this study, the most frequent false positive cases were some chronic ABMR without morphological activity (g0 ptc0) and some cases with multifocal tubulo-interstitial inflammation such as infections and TCMR. At least in part, these latter cases could have been avoided by pathologists by considering peritubular capillary staining as nonspecific near these areas of tubulo-interstitial inflammation. Considering the deep learning strategy in these cases, their limited number did not allow us, at this stage, to train a specific model to distinguish ABMR from TCMR and/or infection biopsies, that would have been optimal for model performance. As for the chronic ABMR cases, it could either represent true false positives, or evolving endothelial injuries without morphological microvascular inflammation, which could require additional molecular studies to settle. False negative cases were mainly due to staining judged as either too focal and/or too weak by pathologists.

Our study has some limitations. Firstly, although including relevant differential diagnoses encountered in kidney transplantation, this immunohistochemical study was performed on a small-scale and single-center cohort. This explains why performance displayed a quite high dispersion (standard deviation), as for example, a difference in classification of a single ABMR case could change the performance of about 6%. Further studies on large-scale unselected cohort will be required to have a more accurate estimate of the performance of these antibodies in ABMR diagnosis. Secondly, other kidney compartments than endothelial cells were obviously stained during some superimposed polymorphic tissue injuries such as tubulo-interstitial inflammation and acute tubular injuries. Sometimes such stains restricted microcirculation analysis to the glomerular compartment, especially with TYMP, in cases with severe tubulo-interstitial inflammation with de facto “uninterpretable” peritubular capillaries. This finding could limit their potential as immunomarker despite, in this cohort, such stains did not significantly lead to false negatives. Thirdly, the thrombotic microangiopathy cases showed more chronic than acute features, which could have favored markers negativity. Fourthly, considering the deep learning analysis, we cannot conclude about the generalization of the displayed models’ performance. Indeed, the small-scale status of the cohort did not allow us to assess performance in an independent holdout set, which would have been optimal to ensure a robust evaluation. Still, internal validation was performed using a threefold cross-validation to ensure at least an honest estimate. Moreover, as already mentioned, the aims of the deep learning analysis were to support pathological findings with an unbiased approach for interpretation and to assess the suitability of such strategies for future studies rather than deploying a turnkey model. Finally, the histological scores of the Banff classification, with their own limitations, were used for inclusion rather than an external standard such as validated molecular classifiers. Future studies should assess unselected cohorts of kidney allograft biopsies, to better reflect the inter-individual heterogeneity of routine cases, allow a better estimate of the immunomarkers’ performance and focus on molecular-classified cases where C4d is non-indicative of an ABMR process (C4d negative ABMR, isolated C4d).

To conclude, this study displays the immunohistochemical expression profile of three interferon-related proteins, WARS1, TYMP and GBP1 in kidney transplantation. We highlighted a singular expression pattern of microcirculation staining in antibody-mediated rejection, revealed by both nephropathologists and a deep learning-based strategy, and deemed to reflect interferon-related endothelial stress during ABMR. This pattern displayed promising diagnostic value in a selected cohort, especially in C4d negative ABMR cases, one of the blind spots of the current Banff classification when no DSA is detectable. Future studies should specifically assess these antibodies in this context.

## Supplementary Information

Supplementary Information.

## Data Availability

The code developed for model training is freely accessible, with user instructions, at https://github.com/bertrandchauveau/GlomProt-IHC. Whole slide images cannot be made publicly available due to regulatory reasons.

## Abbreviations

ABMR: Antibody-mediated rejection
AUC: Area under the receiver operating characteristic curve
CNN: Convolutional neural network
DSA: Donor-specific antibodies
Grad-CAM: Gradient-weighted class activation mapping
HLA: Human leukocyte antigens
Se: Sensitivity
Sp: Specificity
TCMR: T cell-mediated rejection
