# Supplementary material for: WARS1, TYMP and GBP1 display a distinctive microcirculation pattern by immunohistochemistry during antibody-mediated rejection in kidney transplantation
Source: Sci Rep. 2022 Nov 9;12:19094. doi: 10.1038/s41598-022-23078-z (PMC9646783; doi:10.1038/s41598-022-23078-z)
Supplement: Supplementary file 1 — Supplementary Information. [file 41598_2022_23078_MOESM1_ESM.pdf]

Supplementary Information

WARS1, TYMP and GBP1 display a distinctive microcirculation pattern by immunohistochemistry during antibody-mediated rejection in kidney transplantation

Bertrand Chauveau, Antoine Garric, Sylvaine Di Tommaso, Anne-Aurélie Raymond, Jonathan Visentin, Agathe Vermorel, Nathalie Dugot-Senant, Julie Déchanet-Merville, Jean-Paul Duong Van Huyen, Marion Rabant, Lionel Couzi, Frédéric Saltel, Pierre Merville

TABLE OF CONTENTS

Supplemental Table S1. Interpretation results with the WARS1 antibody by immunohistochemistry in the diagnosis of active antibody-mediated rejection

Supplemental Table S2. Interpretation results with the TYMP antibody by immunohistochemistry in the diagnosis of active antibody-mediated rejection

Supplemental Table S3. Interpretation results with the GBP1 antibody by immunohistochemistry in the diagnosis of active antibody-mediated rejection

Supplemental Table S4. Inter-observer reliability with the WARS1, TYMP, GBP1 antibodies in the diagnosis of active ABMR by immunohistochemistry

Supplemental Table S5. Performance of the microcirculation staining with WARS1, TYMP and GBP1 antibodies by immunohistochemistry for predicting ABMR in a selected cohort of kidney biopsies depending on C4d status

Supplemental Table S6. Description of the performance of the deep learning-based approach for each antibody and each iteration of the cross-validation

Supplemental Table S7. Correlations of the deep learning predictions for ABMR with the Banff scores

Supplemental Figure S1. Immunostains with WARS1, TYMP and GBP1 in a T-cell mediated rejection case, original magnification x200.

Supplemental Figure S2. Immunostains with WARS1, TYMP and GBP1 in a chronic antibody-mediated rejection case, without activity according to the 2017 Banff classification, especially with no microvascular inflammation (g0 ptc0), original magnification x200

Supplemental Figure S3. Illustrative morphological patterns of (A) WARS1, (B) TYMP and (C) GBP1 associated by the convolutional neural network with the “Other diagnosis” class (Gradient-weighted Class Activation Mapping, Grad-CAM, approach).

Supplemental Table S1. Interpretation results with the WARS1 antibody by immunohistochemistry in the diagnosis of active antibody-mediated rejection

Diagnosis	Number of total cases (n=53)	WARS1 positivity for active ABMR<br>Number of cases considered positive, n (%)			
		Pathologist 1	Pathologist 2	Pathologist 3	Pathologist 4
**Active ABMR**	16	12 (75)	11 (69)	15 (94)	13 (81)
**Other diagnosis**	37	5 (14)	6 (16)	9 (24)	8 (22)
Including:					
**Non-active cABMR**	5	2 (40)	2 (40)	2 (40)	2 (40)
**Isolated C4d**	3	1 (33)	1 (33)	2 (67)	1 (33)
**SG ABO incompatible**	3	0 (0)	0 (0)	0 (0)	0 (0)
**Non-humoral TMA**	5	0 (0)	0 (0)	0 (0)	0 (0)
**Acute TCMR**	6	1 (17)	0 (0)	2 (33)	2 (33)
**Infection (PVN, APN)**	5	1 (20)	1 (20)	2 (40)	2 (40)
**ATI**	5	0 (0)	1 (20)	0 (0)	1 (20)
**Recurrent GN**	5	0 (0)	1 (20)	1 (20)	0 (0)

Other diagnosis refers to all included differential diagnoses in the cohort, as detailed below. Abbreviations: ABMR, antibody-mediated rejection; WARS1, tryptophan--tRNA ligase, cytoplasmic; cABMR, chronic antibody-mediated rejection; SG, stable graft, TMA, thrombotic microangiopathy; TCMR, T-cell mediated rejection; PVN, polyomavirus nephropathy; APN, acute pyelonephritis; ATI, acute tubular injuries; GN, glomerulonephritis.

Supplemental Table S2. Interpretation results with the TYMP antibody by immunohistochemistry in the diagnosis of active antibody-mediated rejection

Diagnosis	Number of total cases (n=52)	TYMP positivity for active ABMR<br>Number of cases considered positive, n (%)			
		Pathologist 1	Pathologist 2	Pathologist 3	Pathologist 4
**Active ABMR**	16	14 (88)	12 (75)	15 (94)	15 (94)
**Other diagnosis**	36	5 (14)	4 (11)	8 (22)	4 (11)
Including:					
**Non-active cABMR**	5	2 (40)	2 (40)	2 (40)	2 (40)
**Isolated C4d**	3	1 (33)	1 (33)	2 (66)	2 (66)
**SG ABO incompatible**	3	0 (0)	0 (0)	1 (33)	0 (0)
**Non-humoral TMA**	5	0 (0)	0 (0)	1 (20)	0 (0)
**Acute TCMR**	6	2 (33)	0 (0)	0 (0)	0 (0)
**Infection (PVN, APN)**	4	0 (0)	0 (0)	0 (0)	0 (0)
**ATI**	5	0 (0)	0 (0)	1 (20)	0 (0)
**Recurrent GN**	5	0 (0)	1 (20)	1 (20)	0 (0)

Other diagnosis refers to all included differential diagnoses in the cohort, as detailed below. Abbreviations: ABMR, antibody-mediated rejection; TYMP, thymidine phosphorylase; cABMR, chronic antibody-mediated rejection; SG, stable graft, TMA, thrombotic microangiopathy; TCMR, T-cell mediated rejection; PVN, polyomavirus nephropathy; APN, acute pyelonephritis; ATI, acute tubular injuries; GN, glomerulonephritis.

Supplemental Table S3. Interpretation results with the GBP1 antibody by immunohistochemistry in the diagnosis of active antibody-mediated rejection

Diagnosis	Number of total cases (n=52)	GBP1 positivity for active ABMR<br>Number of cases considered positive, n (%)			
		Pathologist 1	Pathologist 2	Pathologist 3	Pathologist 4
**Active ABMR**	15	9 (60)	10 (67)	8 (53)	9 (60)
**Other diagnosis**	37	5 (14)	4 (11)	3 (8)	3 (8)
Including:					
**Non-active cABMR**	5	2 (40)	1 (20)	1 (20)	1 (20)
**Isolated C4d**	3	1 (33)	1 (33)	1 (33)	1 (33)
**SG ABO incompatible**	3	0 (0)	0 (0)	0 (0)	0 (0)
**Non-humoral TMA**	5	0 (0)	0 (0)	0 (0)	0 (0)
**Acute TCMR**	6	1 (17)	0 (0)	0 (0)	0 (0)
**Infection (PVN, APN)**	5	1 (20)	0 (0)	1 (20)	1 (20)
**ATI**	5	0 (0)	1 (20)	0 (0)	0 (0)
**Recurrent GN**	5	0 (0)	1 (20)	0 (0)	0 (0)

Other diagnosis refers to all included differential diagnoses in the cohort, as detailed below. Abbreviations: ABMR, antibody-mediated rejection; GBP1, guanylate-binding protein 1; cABMR, chronic antibody-mediated rejection; SG, stable graft, TMA, thrombotic microangiopathy; TCMR, T-cell mediated rejection; PVN, polyomavirus nephropathy; APN, acute pyelonephritis; ATI, acute tubular injuries; GN, glomerulonephritis.

Supplemental Table S4. Inter-observer reliability with the WARS1, TYMP, GBP1 antibodies in the diagnosis of active ABMR by immunohistochemistry

Kappa value for the WARS1 antibody	Pathologist 4	Pathologist 3	Pathologist 2
**Pathologist 1**	0.837	0.727	0.567
**Pathologist 2**	0.429	0.492	
**Pathologist 3**	0.808		
**Kappa value for the TYMP antibody**	**Pathologist 4**	**Pathologist 3**	**Pathologist 2**
**Pathologist 1**	0.751	0.603	0.700
**Pathologist 2**	0.785	0.718	
**Pathologist 3**	0.841		
**Kappa value for the GBP1 antibody**	**Pathologist 4**	**Pathologist 3**	**Pathologist 2**
**Pathologist 1**	0.693	0.738	0.511
**Pathologist 2**	0.591	0.738	
**Pathologist 3**	0.833		

Cohen's Kappa was calculated for estimation of inter-observer reliability. Abbreviations: ABMR, antibody-mediated rejection; WARS1, tryptophan--tRNA ligase, cytoplasmic; TYMP, thymidine phosphorylase; GBP1, guanylate-binding protein 1.

Supplemental Table S5. Performance of the microcirculation staining with WARS1, TYMP and GBP1 antibodies by immunohistochemistry for predicting ABMR in a selected cohort of kidney biopsies depending on C4d status

	**ABMR cases, total number 16 cases**					
	**WARS1**		**TYMP**		**GBP1**	
	**C4d positive cases, n=5, *n* (%)**	**C4d negative cases, n=11, *n* (%)**	**C4d positive cases, n=4, *n* (%)**	**C4d negative cases, n=12, *n* (%)**	**C4d positive cases, n=4, *n* (%)**	**C4d negative cases, n=11, *n* (%)**
**Pathologist 1**	5 (100)	7 (64)	3 (75)	11 (92)	2 (50)	7 (64)
**Pathologist 2**	3 (60)	8 (73)	2 (50)	10 (83)	1 (25)	9 (82)
**Pathologist 3**	5 (100)	10 (91)	4 (100)	11 (92)	1 (25)	7 (64)
**Pathologist 4**	5 (100)	8 (73)	4 (100)	11 (92)	2 (50)	7 (64)
**Majority rule**	5 (100)	7 (64)	3 (75)	11 (92)	1 (25)	6 (55)

Each antibody was interpreted as positive when a microcirculation staining pattern was observed and negative otherwise. To summarize the results, a majority rule was applied on pathological interpretations, where each case was classified according to the report of most pathologists. In case of ties, the interpretation of the pathologist B.C. was retained. Variations in total number of cases are due to insufficient remaining material for interpretation. Abbreviations: ABMR, antibody-mediated rejection; WARS, tryptophan--tRNA ligase, cytoplasmic; TYMP, thymidine phosphorylase; GBP1, guanylate-binding protein 1.

Supplemental Table S6. Description of the performance of the deep learning-based approach for each antibody and each iteration of the cross-validation

Antibody	Random state	Fold of the cross-validation	AUC of model 1 on the validation set	AUC of model 2 on the validation set, mean (SD)	Sensitivity, mean (SD)	Specificity, mean (SD)	Number of ABMR cases	Number of Other diagnoses
WARS1	42	All	0.737 (0.086)	0.863 (0.112)	0.781 (0.189)	0.899 (0.034)	16	37
WARS1	7	All	0.765 (0.158)	0.902 (0.09)	0.875 (0.103)	0.91 (0.085)	16	37
WARS1	813	All	0.788 (0.041)	0.874 (0.108)	0.845 (0.154)	0.913 (0.139)	16	37
WARS1	1990	All	0.818 (0.14)	0.901 (0.094)	0.822 (0.154)	0.951 (0.048)	16	37
WARS1	39	All	0.776 (0.132)	0.902 (0.09)	0.875 (0.101)	0.946 (0.115)	16	37
TYMP	42	All	0.703 (0.137)	0.798 (0.053)	0.751 (0.075)	0.846 (0.078)	16	36
TYMP	7	All	0.659 (0.062)	0.828 (0.109)	0.809 (0.146)	0.874 (0.07)	16	36
TYMP	813	All	0.675 (0.102)	0.738 (0.196)	0.754 (0.221)	0.821 (0.196)	16	36
TYMP	1990	All	0.722 (0.129)	0.818 (0.167)	0.828 (0.155)	0.802 (0.185)	16	36
TYMP	39	All	0.742 (0.083)	0.826 (0.072)	0.708 (0.067)	0.832 (0.072)	16	36
GBP1	42	All	0.875 (0.038)	0.928 (0.067)	0.899 (0.102)	0.933 (0.015)	15	37
GBP1	7	All	0.784 (0.045)	0.869 (0.149)	0.933 (0.115)	0.767 (0.159)	15	37
GBP1	813	All	0.862 (0.039)	0.927 (0.077)	0.933 (0.115)	0.879 (0.043)	15	37
GBP1	1990	All	0.841 (0.048)	0.898 (0.077)	0.82 (0.13)	0.87 (0.101)	15	37
GBP1	39	All	0.791 (0.06)	0.838 (0.04)	0.823 (0.118)	0.847 (0.107)	15	37
**Details**								
WARS1	42	CV1	0.75	0.891 (0.016)	0.853 (0.055)	0.90 (0.038)	6/16	12/37
WARS1	42	CV2	0.644	0.739 (0.014)	0.626 (0.070)	0.862 (0.067)	7/16	11/37
WARS1	42	CV3	0.816	0.959 (0.010)	1.0 (0.00)	0.929 (0.00)	3/16	14/37
WARS1	7	CV1	0.763	0.881 (0.005)	0.857 (0)	0.836 (0.037)	7/16	11/37
WARS1	7	CV2	0.608	0.824 (0.012)	0.8 (0)	0.882 (0.039)	5/16	13/37
WARS1	7	CV3	0.924	1 (0)	1 (0)	1 (0)	4/16	13/37
WARS1	813	CV1	0.759	0.995 (0.008)	1 (0)	0.976 (0.037)	4/16	14/37
WARS1	813	CV2	0.77	0.838 (0.007)	0.827 (0.06)	0.731 (0.06)	9/16	9/37
WARS1	813	CV3	0.835	0.788 (0.042)	0.693 (0.091)	0.966 (0.079)	3/16	14/37
WARS1	1990	CV1	0.676	0.812 (0.012)	0.693 (0.062)	0.91 (0.023)	6/16	12/37
WARS1	1990	CV2	0.957	1 (0)	1 (0)	1 (0)	3/16	15/37
WARS1	1990	CV3	0.819	0.89 (0.007)	0.857 (0)	0.926 (0.053)	7/16	10/37
WARS1	39	CV1	0.72	0.882 (0.006)	0.8 (0)	1 (0)	5/16	13/37
WARS1	39	CV2	0.681	0.824 (0.012)	0.875 (0)	0.8 (0)	8/16	10/37
WARS1	39	CV3	0.927	1 (0)	1 (0)	1 (0)	3/16	14/37
TYMP	39	CV1	0.584	0.808 (0.007)	0.8 (0)	0.778 (0.04)	5/16	12/36

TYMP	39	CV2	0.853	0.845 (0.007)	0.8 (0)	0.93 (0.025)	5/16	13/36
TYMP	39	CV3	0.673	0.74 (0.03)	0.67 (0)	0.82 (0.013)	6/16	11/36
TYMP	42	CV1	0.73	0.904 (0.006)	1 (0)	0.85 (0.036)	3/16	14/36
TYMP	42	CV2	0.632	0.703 (0.011)	0.71 (0)	0.83 (0)	7/16	12/36
TYMP	42	CV3	0.614	0.876 (0.017)	0.83 (0)	0.96 (0.061)	6/16	10/36
TYMP	7	CV1	0.793	0.919 (0.011)	1 (0)	0.84 (0.037)	6/16	11/36
TYMP	7	CV2	0.607	0.529 (0.033)	0.58 (0.169)	0.61 (0.127)	7/16	12/36
TYMP	7	CV3	0.626	0.765 (0.02)	0.67 (0)	1 (0)	3/16	13/36
TYMP	813	CV1	0.866	1 (0)	1 (0)	1 (0)	4/16	13/36
TYMP	813	CV2	0.683	0.781 (0.046)	0.85 (0.131)	0.72 (0.057)	6/16	13/36
TYMP	813	CV3	0.617	0.672 (0.008)	0.69 (0.058)	0.65 (0.074)	6/16	10/36
TYMP	1990	CV1	0.832	0.82 (0.03)	0.76 (0.035)	0.77 (0.022)	4/16	13/36
TYMP	1990	CV2	0.668	0.757 (0.018)	0.65 (0.049)	0.81 (0.067)	8/16	10/36
TYMP	1990	CV3	0.726	0.9 (0.014)	0.77 (0.06)	0.91 (0.041)	4/16	13/36
GBP1	39	CV1	0.907	0.953 (0)	1 (0)	0.938 (0)	2/15	16/37
GBP1	39	CV2	0.883	0.98 (0.006)	0.935 (0.063)	0.947 (0.056)	8/15	9/37
GBP1	39	CV3	0.834	0.852 (0.009)	0.8 (0)	0.917 (0)	5/15	12/37
GBP1	42	CV1	0.819	0.947 (0.008)	1 (0)	0.833 (0)	6/15	12/37
GBP1	42	CV2	0.733	0.698 (0.005)	0.8 (0)	0.583 (0)	5/15	12/37
GBP1	42	CV3	0.8	0.963 (0.009)	1 (0)	0.877 (0.044)	4/15	13/37
GBP1	7	CV1	0.889	0.976 (0.008)	1 (0)	0.918 (0.018)	5/15	13/37
GBP1	7	CV2	0.817	0.838 (0.009)	0.8 (0)	0.883 (0.041)	5/15	12/37
GBP1	7	CV3	0.881	0.967 (0)	1 (0)	0.833 (0)	5/15	12/37
GBP1	813	CV1	0.814	0.855 (0.049)	0.757 (0.109)	0.924 (0.046)	7/15	11/37
GBP1	813	CV2	0.813	0.852 (0.004)	0.8 (0)	0.752 (0.012)	5/15	12/37
GBP1	813	CV3	0.896	0.987 (0.003)	1 (0)	0.929 (0)	3/15	14/37
GBP1	1990	CV1	0.846	0.879 (0.02)	0.8 (0)	0.97 (0.04)	5/15	12/37
GBP1	1990	CV2	0.801	0.836 (0.012)	0.97 (0.082)	0.769 (0)	4/15	13/37
GBP1	1990	CV3	0.726	0.799 (0.009)	0.743 (0.084)	0.808 (0.124)	6/15	12/37

A deep learning approach was used to build models for the binary classification ABMR *versus* Other diagnosis for each antibody. Two models were trained for a sequential classification. Firstly, a convolutional neural network (Resnet50V2) was trained at the tile level and secondly a random forest classifier was trained at the patient level (*i.e.* whole slide image), based on the output of model 1 for all tiles of the considered patient. Internal validation was performed for the evaluation of models' performance, using 5 iterations (random states) of a 3-fold cross-validation. Average results of each iteration on the validation set are displayed. Abbreviations: ABMR, antibody-mediated rejection; WARS1, tryptophan--tRNA ligase, cytoplasmic; TYMP, thymidine phosphorylase; GBP1, guanylate-binding protein 1; Se, sensitivity; Sp, specificity; AUC, area under the receiver operating characteristic curve; P, Pathologist.

Supplemental Table S7. Correlations of the deep learning predictions for ABMR with the Banff scores

	**Spearman's rank correlation**					
	**WARS1**		**TYMP**		**GBP1**	
**Banff scores**	**rho**	**p-value**	**rho**	**p-value**	**rho**	**p-value**
g + ptc	**0.65**	**1.79E-07**	**0.44**	**9.98E-04**	**0.72**	**1.94E-09**
ptc	**0.63**	**4.15E-07**	**0.42**	**2.04E-03**	**0.69**	**1.76E-08**
g	**0.54**	**3.41E-05**	**0.42**	**1.73E-03**	**0.64**	**3.11E-07**
t-IFTA	**0.39**	**4.11E-03**	**0.42**	**1.80E-03**	**0.38**	**5.17E-03**
ti + i-IFTA + t-IFTA	**0.36**	**8.61E-03**	**0.42**	**2.08E-03**	**0.34**	**1.34E-02**
ti	**0.3**	**2.75E-02**	**0.37**	**6.74E-03**	**0.3**	**3.10E-02**
i-IFTA	**0.28**	**4.55E-02**	**0.31**	**2.42E-02**	0.25	6.95E-02
i + t	**0.28**	**4.16E-02**	**0.36**	**8.78E-03**	**0.37**	**6.22E-03**
i	0.24	8.26E-02	**0.31**	**2.63E-02**	**0.31**	**2.62E-02**
t	0.24	8.81E-02	**0.34**	**1.41E-02**	**0.28**	**4.16E-02**
cg	0.07	6.31E-01	0.01	9.46E-01	0.15	3.05E-01
ah	0.07	6.36E-01	-0.01	9.42E-01	0.18	1.97E-01
mm	0.01	9.50E-01	-0.03	8.37E-01	0.05	7.00E-01
c4d	0	9.99E-01	0.03	8.42E-01	0	9.76E-01
cv	-0.01	9.41E-01	-0.11	4.65E-01	0.16	2.70E-01
ci	-0.06	6.68E-01	0.01	9.62E-01	-0.1	4.60E-01
ct	-0.06	6.68E-01	0.01	9.62E-01	-0.1	4.60E-01
v						

Prediction values of model 2 from the validation sets were used for the correlations with the Banff scores. For each antibody, prediction values from the 3 folds of the cross-validation were normalized from 0 to 1 with the same threshold value (0.5). Statistical significance is highlighted using bold font. No cases with arteritis (v > 0) were present in this cohort, therefore no correlations could be computed. Banff scores are arranged in descending order of the correlations with the WARS1 antibody. Abbreviations: ABMR, antibody-mediated rejection.

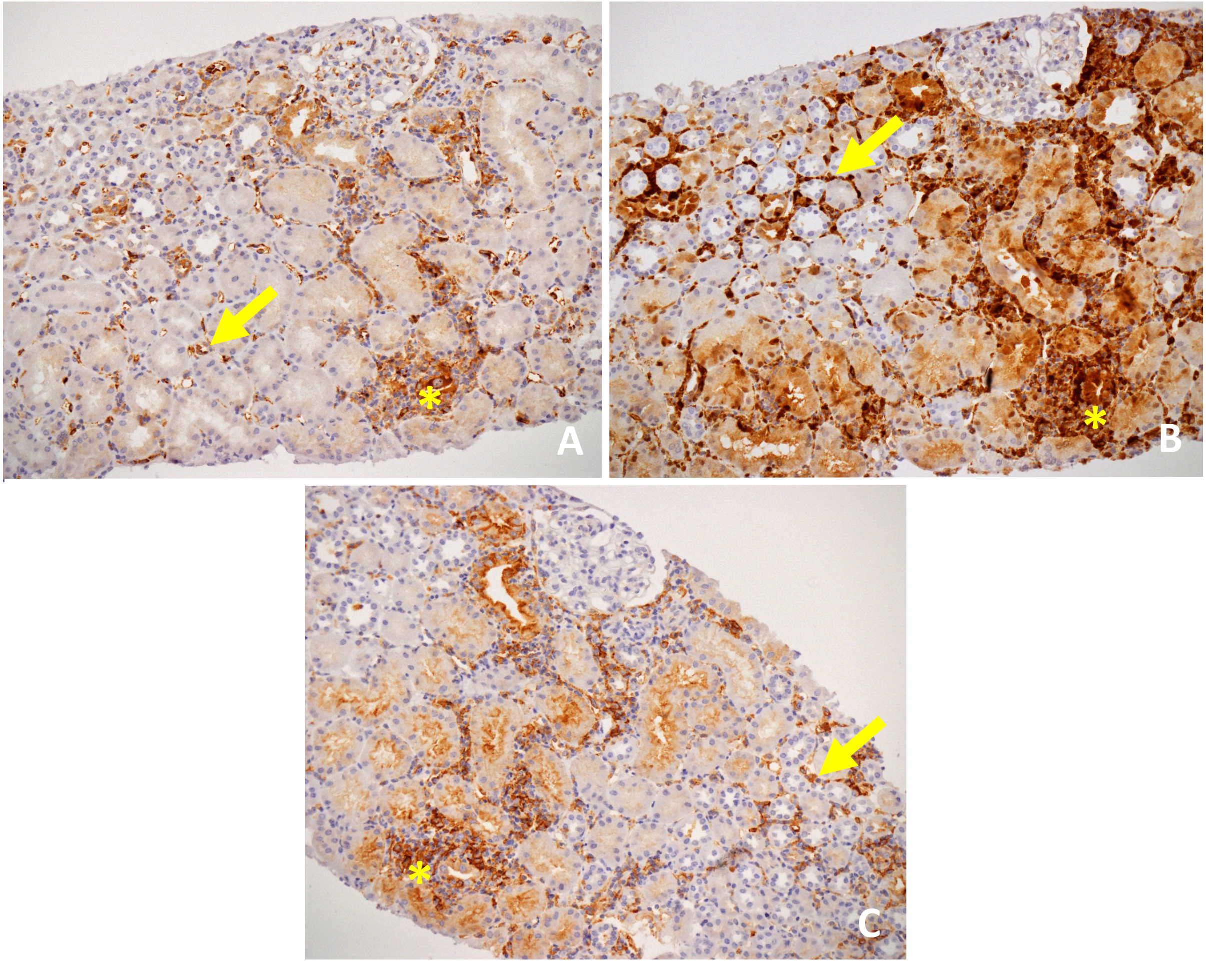

Supplemental Figure S1. Immunostains with WARS1, TYMP and GBP1 in a T-cell mediated rejection case, original magnification x200. (A) WARS1 staining. Nearby an interstitial inflammatory infiltrate (asterisk), endothelial cells of peritubular capillaries are moderately stained (arrow), reminiscent of a microcirculation pattern. Endothelial positivity in the glomerulus is similar to constitutive staining. This case was misclassified by 3 pathologists. (B) TYMP staining. While there is a strong tubulo-interstitial positivity (asterisk), including tubular, inflammatory and endothelial cells, adjacent peritubular capillaries are also strongly stained (arrow). The glomerulus showed no specific endothelial positivity. This case was misclassified by one pathologist. (C) GBP1 staining. Similarly, a moderate to strong staining of tubular and inflammatory cells is seen (asterisk). Some nearby endothelial cells of peritubular capillaries are also moderately stained (arrow), reminiscent of a microcirculation pattern. The glomerulus is negative. This case was misclassified by one pathologist. Please note that here, only one field is displayed and the assessment of the whole biopsy is needed for classification.

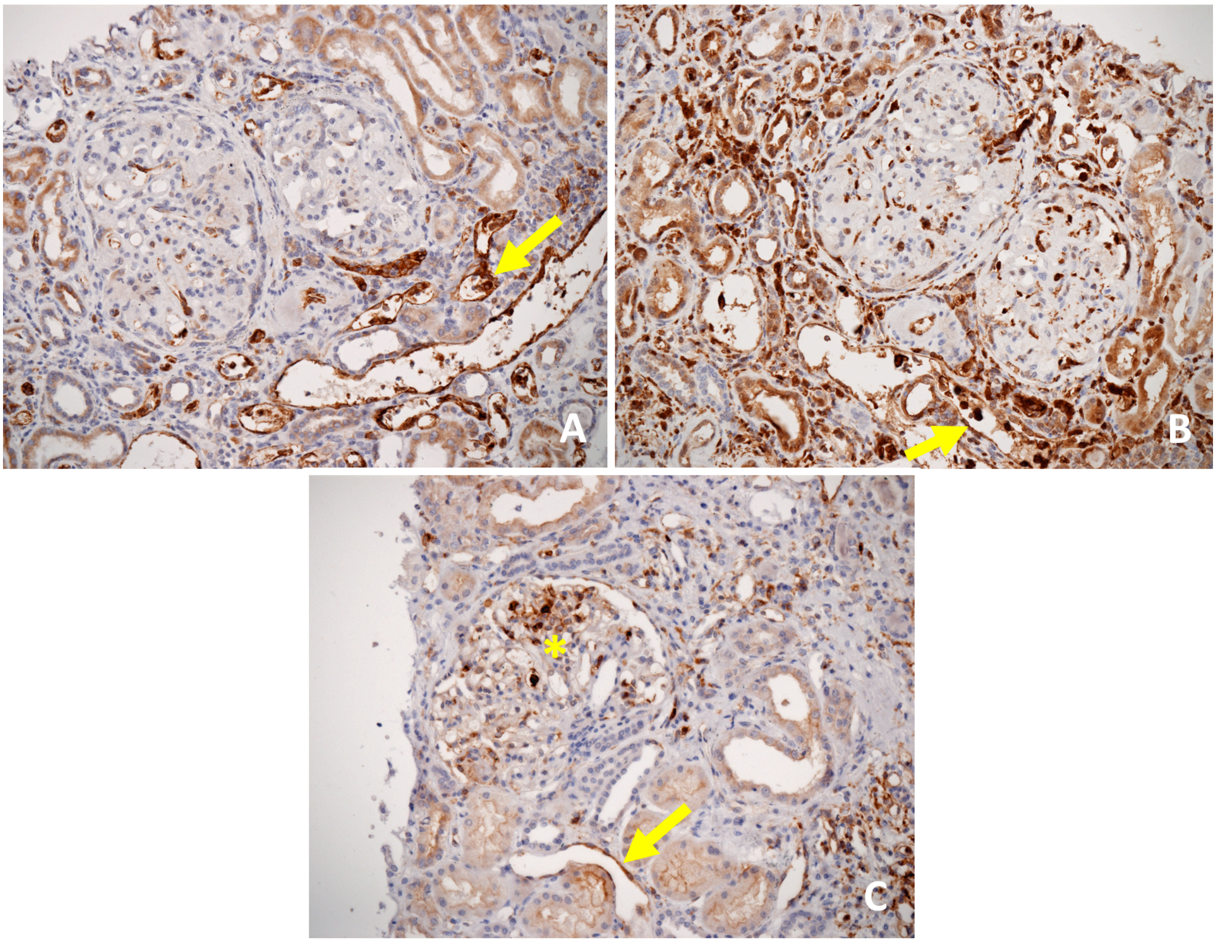

Supplemental Figure S2. Immunostains with WARS1, TYMP and GBP1 in a chronic antibody-mediated rejection case, without activity according to the 2017 Banff classification, especially with no microvascular inflammation (g0 ptc0), original magnification x200. (A) WARS1 staining. Note the strong endothelial positivity on peritubular capillaries (arrow), that prompted pathologists to a misclassification as active antibody-mediated rejection (ABMR). Positivity of the glomeruli is sparse. A weak and diffuse positivity is observed in tubular cells. (B) TYMP staining. A strong positivity is seen in inflammatory cells as well as a moderate expression in tubular cells, that makes it difficult to assess endothelial positivity, which is moderate in peritubular capillaries (arrow) and weak in the glomeruli. This case was misclassified by one pathologist. (C) GBP1 staining. A globally, moderate and segmental staining is visible in the glomerulus (asterisk), on some endothelial and inflammatory cells. In the peritubular capillaries, a moderate and sparse staining is seen on some endothelial cells (arrow). This case was misclassified by one pathologist.

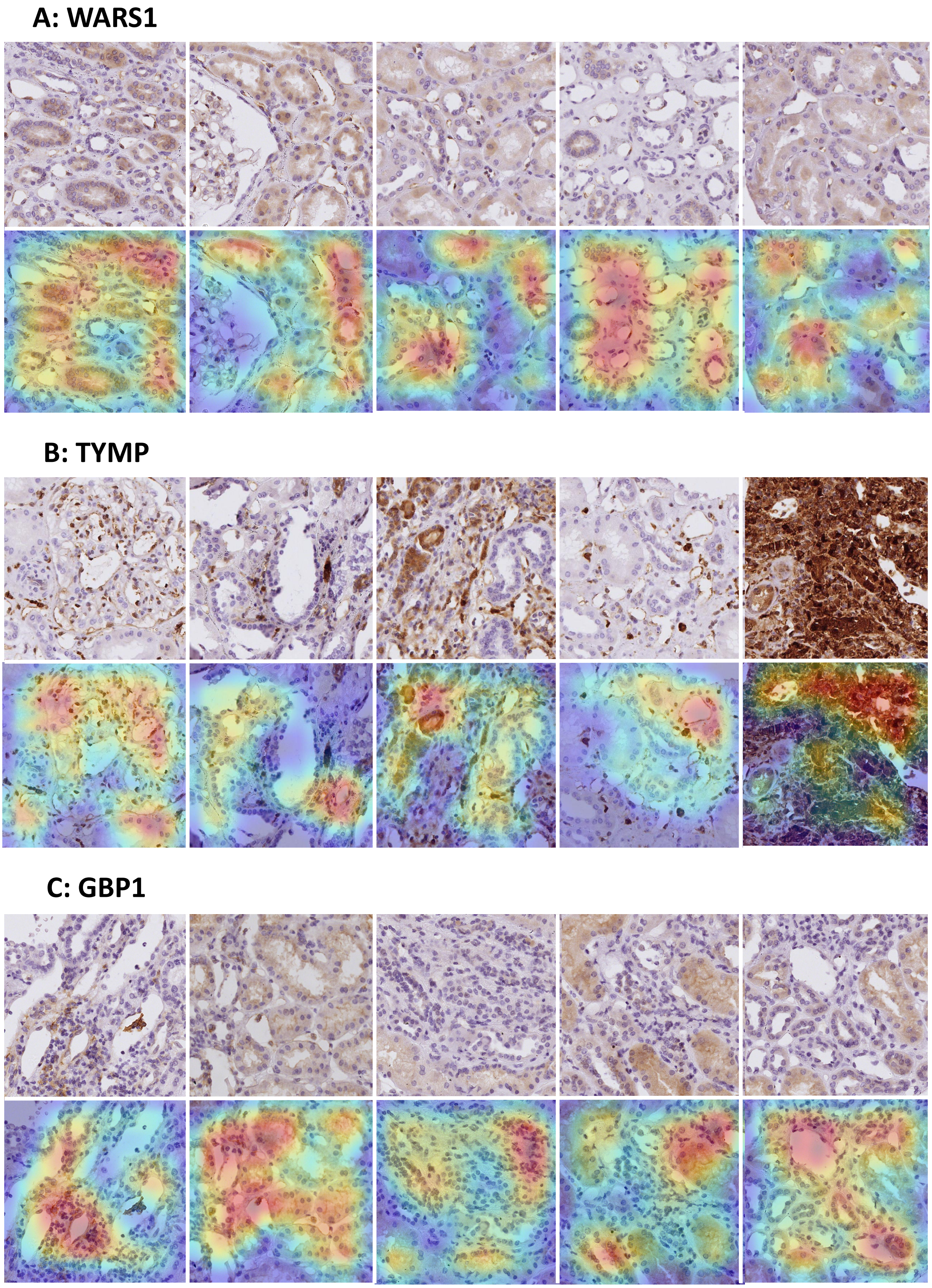

Supplemental Figure S3. Illustrative morphological patterns of (A) WARS1, (B) TYMP and (C) GBP1 associated by the convolutional neural network with the “Other diagnosis” class (Gradient-weighted Class Activation Mapping, Grad-CAM, approach). For each antibody, 5 tiles (512 x 512 pixels) among the most associated with the “Other diagnosis” class are displayed, with the corresponding heatmap

presented below (from blue to red, where red represents regions of utmost importance by the neural network for class consideration). Notice that, for each antibody, the neural network focused on circular structures lined by unstained (or weakly stained) flat cells, thus frequently corresponding to peritubular capillaries or medullary vasa recta, more rarely to glomerular capillaries, and sometimes mistakenly to atrophic tubules or thin limbs of Henle's loops.
